# Fabrication of Core-Shell PEI/pBMP2-PLGA Electrospun Scaffold for Gene Delivery to Periodontal Ligament Stem Cells

**DOI:** 10.1155/2016/5385137

**Published:** 2016-05-26

**Authors:** Qiao Xie, Lie-ni Jia, Hong-yu Xu, Xiang-gang Hu, Wei Wang, Jun Jia

**Affiliations:** ^1^State Key Laboratory of Military Stomatology and National Clinical Research Center for Oral Diseases and Shaanxi Key Laboratory of Oral Diseases, Department of Prosthodontics, School of Stomatology, The Fourth Military Medical University, Xi'an, Shaanxi 710032, China; ^2^No. 422 Hospital of PLA, Zhanjiang, Guangdong 524005, China; ^3^Hospital of PLA No. 93523 Unit, Yongji, Shanxi 044500, China

## Abstract

Bone tissue engineering is the most promising technology for enhancing bone regeneration. Scaffolds loaded with osteogenic factors improve the therapeutic effect. In this study, the bioactive PEI (polyethylenimine)/pBMP2- (bone morphogenetic protein-2 plasmid-) PLGA (poly(D, L-lactic-co-glycolic acid)) core-shell scaffolds were prepared using coaxial electrospinning for a controlled gene delivery to hPDLSCs (human periodontal ligament stem cells). The pBMP2 was encapsulated in the PEI phase as a core and PLGA was employed to control pBMP2 release as a shell. First, the scaffold characterization and mechanical properties were evaluated. Then the gene release behavior was analyzed. Our results showed that pBMP2 was released at high levels in the first few days, with a continuous release behavior in the next 28 days. At the same time, PEI/pBMP2 showed high transfection efficiency. Moreover, the core-shell electrospun scaffold showed BMP2 expression for a much longer time (more than 28 days) compared with the single axial electrospun scaffold, as evaluated by qRT-PCR and western blot after culturing with hPDLSCs. These results suggested that the core-shell PEI/pBMP2-PLGA scaffold fabricated by coaxial electrospinning had a good gene release behavior and showed a prolonged expression time with a high transfection efficiency.

## 1. Introduction

Periodontal disease involves the deterioration of periodontal bone and is the primary cause of tooth loss in adults. At present, periodontal disease treatments focus on promoting periodontal tissue regeneration by controlling inflammation and formatting new attachment [1–3]. While periodontal treatments such as scaling, surgical cleaning, bone grafts, and guided tissue regeneration are generally successful, the ability to regenerate the damaged tissues predictably still remains a major unmet objective for conventional treatment strategies [4, 5]. Therefore, Langer in 1993 proposed tissue engineering as a possible technique for periodontal tissue regeneration [6, 7]. Tissue engineering mimics the natural healing process to reconstruct or regenerate damaged tissue using three fundamental tools: progenitor cells, signaling molecules, and scaffolds.

Mesenchymal stem cells (MSCs), as multipotent progenitor cells, can be isolated from adult bone marrow or prenatal tissues. As one of the MSCs from dental origin, periodontal ligament stem cells (PDLSCs) have been isolated and tested for their ability to develop into various types of tissues in* in vitro* and* in vivo* studies [8]. Due to this multipotent differentiation ability, PDLSCs can be used in regenerative medicine because they provide a source of cells not only for dental tissue regeneration but also for repair of nondental structures such as bone and nerves [9]. Human PDLSCs (hPDLSCs) have proven stem cell characteristics, including osteogenic-differentiating and immune-modulating properties [10]. More importantly, hPDLSCs can be harvested from medical waste materials such as discarded extracted teeth without additional surgery that may cause patients to experience physical deformity, pain, and considerable expense. Therefore, hPDLSCs are considered a promising cell type for cell-based gene delivery and periodontal tissue engineering applications [11].

Bone regeneration is regulated by various bioactive agents such as platelet derived growth factor (PDGF), transforming growth factor-beta (TGF-*β*), bone morphogenetic proteins (BMPs), and insulin-like growth factor (IGF), which are important factors involved in the osteogenic process. Among the many BMPs, BMP2, a member of the highly conserved transforming growth factor-*β* superfamily, is a potent inducer of osteogenesis and plays an important role in bone formation and remodeling [12–15]. Although successful bone regeneration was induced by BMP2 in preclinical and clinical application, supraphysiological dosage of BMP2 was required, which may cause low efficiency and potentially toxic effect. Thus, a new modality for BMP2 treatment is required to overcome these shortcomings. Gene delivery using various vectors has been suggested as a novel approach, in which target gene materials are injected into and around the wound to infect the host cells, enabling them to produce the therapeutic proteins. This strategy can avoid the disadvantages caused by the direct application of exogenous growth factors such as BMP2 [16]. Genetically modified carriers are mainly divided into viral and nonviral vectors. The drawback of the viral vectors is that they cause a strong body immune response; they are infectious and potentially carcinogenic. In addition, virus carrier preparation is difficult and expensive, and the carrier capacity is small. Despite lower price and virus-free carrier, the traditional nonviral vector, such as liposomes, chitosan, and calcium phosphate, produces less antigenicity, lower transduction efficiency, and shorter expression duration. Thus, it is difficult to obtain a significant gene expression with traditional nonviral vector systems. Compared to other nonviral systems and especially viral vectors, cationic nonviral delivery systems have several advantages such as low toxicity and antigenicity due to only biological lipids composition and long-term expression with less risk of insertional oncogenesis. Polyethylenimine (PEI) is a cationic polymer that can compact the long DNA chains into submicron particles [17–19]. PEI is a potent nonviral transfection agent, which has high transfection activity* in vitro* and moderate activity* in vivo*.

Gene activated matrix (GAM), which combines gene delivery with scaffold has been commonly discussed as a powerful strategy that can provide opportunities to more effectively apply gene delivery for tissue engineering [20, 21]. However, GAM fabrication techniques such as freeze-drying emulsion, solution casting method, leaching, gas foaming method, and thermally induced phase separation method have the limitation of reducing plasmid activity by organic solvent; thus, the gene release behavior is uncontrollable and transfection efficiency is not ideal [8–10]. Electrospinning is a simple method of preparing nanofibers. By altering polymer solution composition and parameters, the mechanical, biological, and kinetic properties of the electrospun nanofibrous scaffold can be easily controlled. Electrospun nanofibrous membrane has reticular structure with high specific surface area, high porosity, and interconnect pores, which is similar to the natural extracellular matrix (ECM) and can enhance the adhesion, proliferation, and growth of cells [22]. Preparation of GAM by common single electrospinning method consists of mixing gradable polymer material with plasmid vector. In this way, the plasmid is inevitably exposed to organic solvents during the electrospinning process, which will reduce its activity. In addition, the sudden release of plasmid from this kind of nanofibers may lead to a short valid time in the initial period. Coaxial electrospinning is an innovative extension of electrospinning, which uses two concentrically aligned capillaries to enforce the formation of the fibers with a core-shell structure, where multiple biomolecules at each layer can be designed to diffuse out sequentially. Differences in the diffusion pathways of gene vectors through two layers composed of different materials can alter the release rates of the incorporated gene vectors in each layer, which have already been observed in many drug delivery studies using core-sheath structures [23–25].

This study aimed to fabricate a core-shell PEI/BMP2 plasmid (pBMP2)-poly(D, L-lactic-co-glycolic acid) (PLGA) electrospun scaffold for gene delivery as GAM by coaxial electrospinning in order to protect pBMP2 from direct exposure to organic solvents and control its release. The characterization and mechanical properties of the GAM were evaluated, and the effects of this nanofibers scaffold loaded with pBMP2 on cell viability and BMP2 expression in hPDLSCs were investigated.

## 2. Materials and Methods

### 2.1. Materials

Poly(D, L-lactic-co-glycolic acid) (PLGA, MW 100,000) with a 75 : 25 monomer ratio (lactic acids : glycolic acids) was provided by Daigang Company (Shandong, China). PEI and 1,1,1,3,3,3-hexafluoro-2-propanol (HFIP) were obtained from Aladdin Industrial Corporation (Shanghai, China). Phosphate-buffered saline (PBS) pH 7.4 containing 0.1 M sodium phosphate and 0.15 M sodium chloride, used for* in vitro* release study, was purchased from Sigma-Aldrich (St. Louis, MO, USA). The plasmid containing human BMP2 and enhanced green fluorescent protein gene (GFP) was generously donated by Cell Engineering Research Center, Fourth Military Medical University (Xi'an, China). Plasmid extraction kit (QIAGEN Plasmid Maxi Kit) was purchased from QIAGEN Company (Germany).

### 2.2. Electrospinning

PLGA was dissolved in HFIP at 12% wt and stirred overnight at room temperature to prepare the sheath solution of nanofibers. The pBMP2 was amplified in the* Escherichia coli* strain DH5*α* and then purified by QIAGEN Plasmid Maxi Kit. Next, the PEI/pBMP2 nanoparticles were created as a result of static attraction between PEI and pBMP2 to form the core solution of core-shell fibers. The size of pBMP2 encapsulated particle was mainly evaluated by N/P ratio [26]. PEI/pBMP2 nanoparticles prepared with an N/P ratio of 10 tended to have higher thermal dynamics ability. pBMP2 solution was prepared at the concentration of 500 *μ*g/mL in 0.1x TE buffer (pH 7.3). Then 2 mL of the pBMP2 aqueous solution and 750 *μ*g of PEI solution (99% purity) were quickly mixed together and stirred for 5 min.

The electrospinning equipment (SH2535, UCALERY Co., Beijing, China) was used to prepare the electrospun core-shell scaffolds. The setting to prepare the core-shell scaffolds consisted of two coaxial syringe pumps with different flow rates. In this study, the PLGA solution was used to form the outer shell and the PEI/pBMP2 solution was used to form the inner core. These two solutions were connected to the coaxial needles, a 16 G (ID = 1.6 mm) outer needle and a 25 G (ID = 0.5 mm) inner needle, respectively, and then concentrically placed. The electrospinning followed the same conditions (applied voltage: 25 kV; 2 mL/h for the sheath flow rate and 0.6 mL/h for the core flow rate; air gap, 15 cm). The electrospinning process lasted for 5 h. Under the same condition, PEI/pBMP2-loaded PLGA blended nanofibers and pure PLGA nanofibers were also fabricated by single axial electrospinning as single axial scaffolds group and PLGA scaffolds group, respectively. The prepared membranes were vacuum-dried for 24 h and stored in a refrigerator at 4°C.

### 2.3. Scaffold Characterization

#### 2.3.1. Morphology Characterization

The surface morphology of the electrospun membrane was observed using HITACHI S-4800 SEM. The samples were mounted on metal stubs and sputter coated with palladium-platinum-gold for 60 s. Images were taken at an accelerating voltage of 5 kV. The fibers diameters and pore sizes were measured using Image-Pro Plus software (*n* = 100).

The samples were placed on the copper meshes directly obtained during coaxial electrospinning process and observed using a JEM-3010 TEM (JEOL Co., Japan), with an accelerating voltage of 300 kV. Bright field images were collected with an 11-megapixel SC1000 Orius CCD camera (Gatan, Inc.). Image analysis was performed using Digital Micrograph (Gatan, Inc.).

#### 2.3.2. Mechanical Properties

The tensile properties of the electrospun membrane were carried out using a universal tensile tester (EZ-SX, Shimadzu, Japan). Different scaffolds with varying compositions and thicknesses were sectioned into 1 cm × 6 cm sections (dog-bone shaped) and tested at a speed of 1 mm/min.

#### 2.3.3. *In Vitro* Release Behavior

The two groups of electrospun scaffolds containing PEI/pBMP2 (the core-shell scaffolds and the single axial scaffolds) were cut into round sections at a diameter of 10 mm, sterilized with 70% ethanol, and washed with PBS, and each section was incubated with 2 mL PBS in Eppendorf tubes placed on a constant-temperature shaker (CHA-S, Guohua, China). No PEI/pBMP2 nanoparticles were present in the pure PLGA scaffolds; thus, we did not test the PLGA release behavior. At scheduled time interval, eluents of each group were obtained and stored at 4°C until the end of the release assay. The amount of released plasmid BMP2 was quantified using the PicoGreen Assay. Solutions were excited at 485 nm wavelength, and emission was measured at 530 nm in a microplate reader (Tecan Infinite 200 PRO, Switzerland). Optical density (OD) value of each sample was recorded. DNA concentration was calculated using the standard curve.

### 2.4. *In Vitro* Study

#### 2.4.1. Cell Culture

Primary hPDLSCs used in this study were generously donated by Cell Engineering Research Center of Fourth Military Medical University (Xi'an, China) and cultured in MEM alpha medium (Hyclone, USA) supplemented with 10% fetal bovine serum (Hyclone, USA) and 1% penicillin/streptomycin. The hPDLSCs were maintained in a humidified incubator at 37°C and 5% CO_2_ and used in the following experiments at passage three or four.

#### 2.4.2. Cell Viability

To evaluate cell viability, three groups of scaffolds were analyzed by MTT assay (Sigma-Aldrich). Cells were seeded into 24-well plates at an initial density of 5 × 10^4^/well with 1 mL growth medium and cultured with the three groups of scaffolds. One cell group was cultured without any scaffold as a blank control. After 1, 3, 5, and 7 days of culture, 100 *μ*L of MTT solution was added to each well and incubated for 4 h. After removing the medium containing the MTT, 1 mL dimethyl sulfoxide (DMSO) (Sigma-Aldrich) was added to each well to extract the formazan crystals under gentle shaking. The absorbance intensities were measured at 595 nm using a microplate reader.

#### 2.4.3. Transfection Efficiency

No PEI/pBMP2 nanoparticles were present in the PLGA scaffolds; thus, we did not test the PLGA transfection efficiency. The control group was designated as an equivalent amount of pBMP2. Gene transfection efficiency was evaluated by GFP expression. The two groups of scaffolds containing PEI/pBMP2 were cut into round sections at a diameter of 10 mm, sterilized with 70% ethanol, washed with PBS, and placed into 6-well plates containing 2 mL PBS. The PBS was replaced by an equal amount of *α*-MEM supplemented with 10% fetal bovine serum and 1% penicillin/streptomycin at 0 d, 2 d, 4 d, 6 d, 13 d, 20 d, and 27 d, and hPDLSCs were seeded in these 6-well plates at the concentration of 3 × 10^5^ cells per well when the PBS was replaced. After 24 h, GFP expression within cells was observed using confocal microscopy (FV1000, OLYMPUS, Japan) and the final transfection efficiency was determined by flow cytometry (FCM) (BD FACS Aria Flow Cytometer, Beckman, USA). A number of 10,000 cells were counted for each sample.

#### 2.4.4. Osteogenic Activity

In order to study the osteogenic activity, BMP2 expression ability was evaluated only in the two PEI/pBMP2-loaded scaffolds groups. The two groups of scaffolds were cut into round sections at a diameter of 10 mm, sterilized with 70% ethanol, washed with PBS, and placed into the wells of 6-well plates. hPDLSCs were seeded in these 6-well plates at a concentration of 3 × 10^5^ cells per well and cultured using the same method as described above. The plated cells were collected at 3 d, 7 d, 14 d, 21 d, and 28 d. The controls were designated as 0 days. The expression of mRNAs was quantitatively detected using DyNamo SYBR Green qPCR kit (Finnzymes, Espoo, Finland). Experiments were repeated three times for each sample. The primer's sequences of the targeted genes are listed in Table 1. Runx2 and BMP2 protein expression were assayed by western blotting. Calcium (Ca) concentration at four weeks was evaluated in the cell lysates from two groups as previously described. Ca concentration was quantitatively detected using calcium ion content assay kit (Sigma-Aldrich).

### 2.5. Statistical Analysis

Quantitative values were averaged and expressed as mean ± standard deviation. Student's *t*-test was used to evaluate the difference between experimental groups and *P* < 0.05 was considered statistically significant. The SPSS statistical software (IBM; version 20.0) was used for data analysis.

## 3. Results

### 3.1. Characterization of Nanofibrous Scaffolds

#### 3.1.1. Morphological Characterization

**Figure 1:**
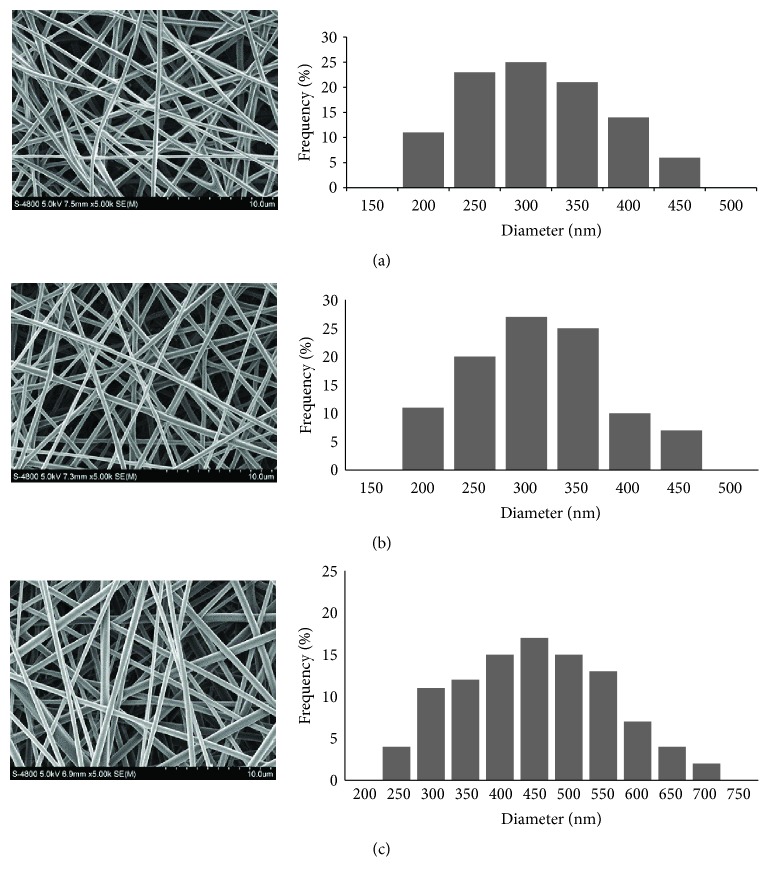
Morphology of electrospun scaffolds. (a) PLGA nanofibers. (b) Single axial nanofibers. (c) Core-shell nanofibers.

As shown in Figure 1, both the single axial and the core-shell scaffolds were highly porous and had a smooth surface. The average diameters of the PLGA nanofibers, single axial nanofibers, and core-shell nanofibers were 287 ± 63 nm, 297 ± 103 nm, and 481 ± 103 nm, respectively.

**Figure 2:**
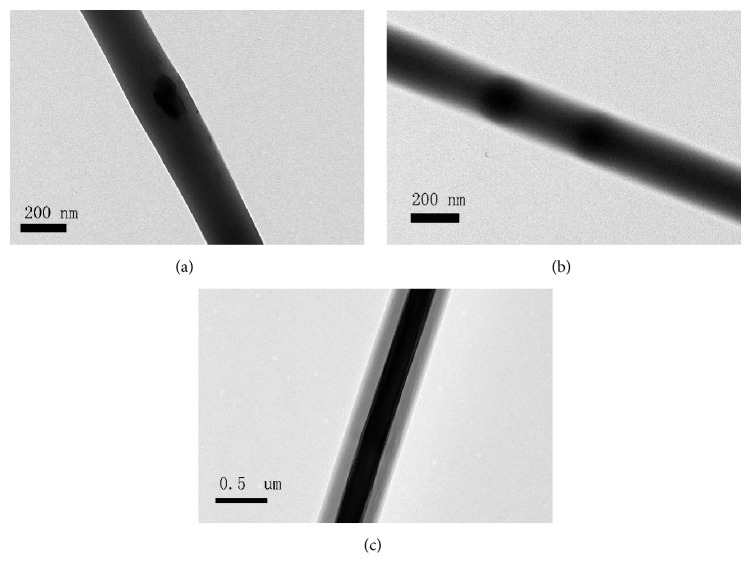
TEM images. (a) PLGA scaffolds. (b) Single axial scaffolds. (c) Core-shell scaffolds.

The interior structure of the composite fibers was investigated by TEM (Figure 2). As we expected, the core-shell nanofibers had a clear core/sheath structure, while the pBMP2 microspheres were randomly dispersed throughout the single axial nanofiber.

#### 3.1.2. Mechanical Properties

Table 2 shows the mechanical properties of the core-shell scaffold. The values were approximately 11 MPa for tensile modulus, 29% ultimate strain, and 3.5 MPa for ultimate stress, which were slightly lower than those of the PLGA scaffolds (*P* < 0.05). However, there was no significant difference between core-shell scaffold and single axial scaffold group (*P* > 0.05).

#### 3.1.3. *In Vitro *Release Behavior

**Figure 3:**
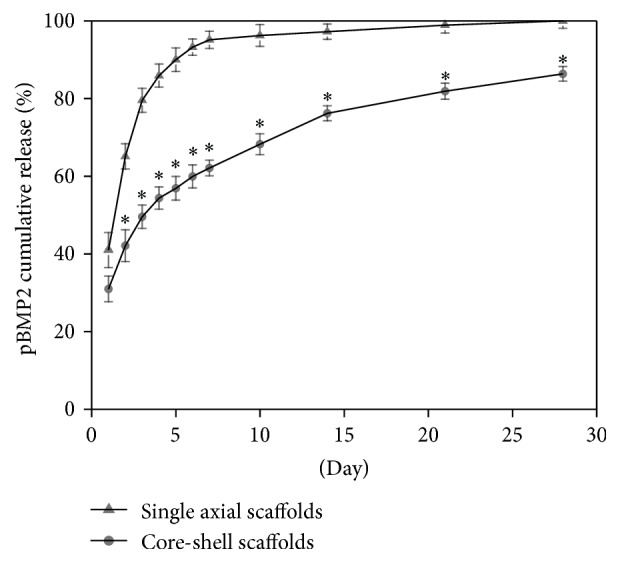
*In vitro* release curves of the single axial scaffolds and core-shell scaffolds at days 1, 2, 3, 4, 5, 6, 7, 10, 14, 21, and 28 (*n* = 3, ^*∗*^ *P* < 0.05).

Figure 3 shows the pBMP2 release behavior of two groups of scaffolds. After a slight burst release occurred within the first 24 h, the pBMP2 release became stable and was sustainable. A sudden release was observed in the first day, with 31.98% and 42% from the core-shell scaffolds and the single axial scaffolds, respectively. Compared with the single axial scaffolds, release of pBMP2 in the core-shell scaffolds increased significantly for 2, 3, 4, 5, 6, 7, 10, 14, 21, and 28 days (*P* < 0.05).

### 3.2. *In Vitro* Studies

#### 3.2.1. Cell Viability

To confirm the biocompatibility, hPDLSCs were cultured with the three groups of scaffolds (the core-shell scaffolds, the single axial scaffolds, and the PLGA scaffolds) compared with the blank control. After culture, the scaffolds with pBMP2 did not show a negative effect on cell proliferation. The optical density value was increased in a time-dependent manner from day 1 to day 7 in all groups. Indeed, the optical density in PEI/pBMP2-loaded scaffolds was increased in the same extent as PLGA scaffolds and blank control (*P* > 0.05).

#### 3.2.2. Transfection Efficiency

To evaluate gene expression efficiency in hPDLSCs, transfection experiments were carried out using GFP as a reporter gene.  Figure 5 shows pBMP2 transfection observed by confocal microscopy during the 7 days. The core-shell scaffolds and the single axial PEI/pBMP2-loaded PLGA scaffolds showed a significant increase in transfection efficiency compared to the control group which only contained the pBMP2 plasmid without PEI. In addition, the core-shell scaffolds showed a large number of GFP-positive cells homogeneously distributed across the electrospun meshes on day 7, while the other groups showed few GFP-positive cells.

The ability of the electrospun fibers to enhance the cell transfection was evaluated by FCM. Transient transfection efficiency in Figure 6 showed a steady time-dependent decrease in both groups, although the core-shell scaffolds showed stable transfection efficiency for a longer time, more than 28 days. Compared to the single axial scaffolds, the core-shell scaffolds had significantly higher transfection efficiency for 7, 14, 21, and 28 days (*P* < 0.05).

#### 3.2.3. Osteogenic Activity

Cbfa1/Runx2 and BMP2 mRNA expression was quantitatively evaluated by real-time PCR. In the core-shell scaffolds group, BMP2 mRNA levels were increased approximately 13-fold more than the control after 3 days. After 7 days, BMP2 mRNA expression was 8.5-fold higher than the control, and it was still 3.5-fold higher than the control after 28 days. The Runx2 levels increased 11-, 8.5-, 6.4-, 4.2-, and 4-fold more than the control on days 3, 7, 14, 21, and 28, respectively. In the hPDLSCs cultured with the single axial scaffolds group, BMP2 gene expression showed a 10- and 5-fold increase, while Runx2 levels showed a 12- and 5.7-fold increase at days 3 and 7, respectively, compared to the control. After approximately 10 days, the BMP2 and Runx2 levels were similar to the control group (Figures 7(a) and 7(b)). The expression of both BMP2 and Runx2 proteins involved in osteogenesis was evaluated by western blot (Figure 7(c)). BMP2 and Runx2 protein expression was increased during the first week, but the single axial scaffolds group showed a clear decrease also during the second week. After 14 days, both proteins expressions were also significant in the core-shell scaffolds. The level of protein expression was consistent with the mRNA expression. The core-shell scaffolds clearly showed a prolonged expression time. Furthermore, the higher Ca concentration was observed in the core-shell scaffolds group than that in the single axial scaffolds group (*P* < 0.05) (Figure 7(d)).

## 4. Discussion

Periodontal tissue consists of four component structures: alveolar bone, periodontal ligament, cementum, and gingiva. Periodontal regeneration is a complex process. Appropriated and environmental signals are needed to activate cells growth and differentiation, usually working in a controlled temporospatial manner. Successful periodontal tissue engineering needs several factors including cells, biodegradable scaffold, and biological factors to mimic the critical aspects of these biological processes. GAM combines gene therapy and tissue engineering to create a novel solution with a great potential for the restoration of structure and function of damaged or dysfunctional tissues. In this study, a core-shell PEI/pBMP2-PLGA electrospun scaffold for gene delivery was fabricated by coaxial electrospinning.

Coaxial electrospinning was firstly demonstrated by Sun et al. [27]. In this study, two solutions, PLGA and PEI/pBMP2, underwent coaxial and simultaneous electrospinning through different feeding capillary channels and flow rates in one needle to generate core-shell composite nanofibers. The structure and morphology of the produced fibers are determined by a synergetic effect of formulation and process parameters. Despite the formation of a stable Taylor cone during fabrication of the fiber meshes for all the formulations, the core-shell fibers showed a greater distribution of diameters as showed in Figure 1. Both these observations suggest that the inclusion of highly charged moieties, such as a cationic gene delivery vector and an anionic plasmid DNA, significantly affect the electrospinning properties of PLGA. pBMP2 can be incorporated in the electrospun nanofibrous structures, as illustrated in Figure 2(b); PEI/pBMP buffers with PLGA polymer solutions in organic solvents, followed by electrospinning the nanofibers. But the core-sheath structures, as showed in Figure 2(c), can protect PEI/pBMP2 from direct exposure to organic solvents and control the release of pBMP2.

The mechanical properties of GAM are important in tissue engineering. As we know, the potential periodontal regeneration scaffold should provide sufficient mechanical stability during the process of tissue regeneration and structural degradation. It also should be easily handled and applied around the affected tooth root and bone. In Table 2, average elongation at break and average tensile strength demonstrated that scaffold groups which incorporated pBMP2 have lower strength than PLGA scaffolds group. Compared to PLGA nanofibers with uniform circular cross-sections, core-shell nanofibers containing PEI/pBMP2 nanoparticles with nonuniform cross-sections showed different ductility. This probably also stems from the diameter increase, the slight decrease in porosity, and the interfusion of PEI/pBMP2 nanoparticles different from PLGA after assembly, which could result in an increase in the modulus. However, in view of the future clinical application, the mechanical strength data of these two incorporated pBMP2 groups are still within the satisfactory limits and comparable to the data of commercially available GTR membranes in the previous papers [28, 29].

To achieve a successful gene delivery, another important aspect that should be considered is to ensure the release of gene within the time frame of tissue regeneration. The sustained release of the plasmid DNA, whose release kinetics was determined by both the contents of the copolymers used to produce the nanofibers and the fiber structure itself [30, 31]. In our study, pBMP2 was incorporated into PEI and then combined with the PLGA by electrospinning for delivery to a target site. To confirm whether pBMP2 can be continuously released from the scaffolds, pBMP2 release profiles were evaluated in the electrospun scaffolds. As showed in Figure 3, 42% pBMP2 was released from the single axial scaffolds and only 31.98% was released from the core-shell scaffolds in the first day. Compared with the single axial scaffolds, release of pBMP2 in the core-shell scaffolds also decreased significantly for 2, 3, 4, 5, 6, 7, 10, 14, 21, and 28 days (*P* < 0.05). It indicated that the core-shell scaffolds can provide an efficient means to control the sequential release of multiple vectors and can simultaneously protect gene vectors in the core-layer against the relatively harsh processes.

**Figure 4:**
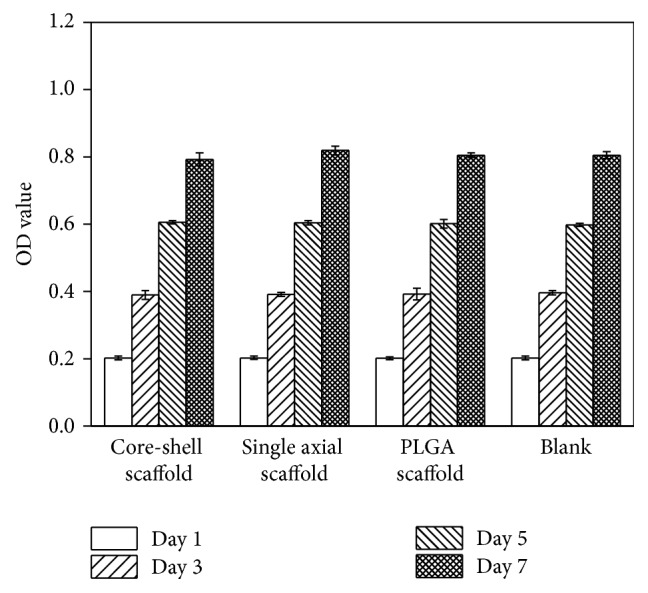
hPDLSCs cultured with core-shell scaffolds, single axial scaffolds, PLGA scaffolds, and tissue culture plates (blank). The OD values of all the groups were increased until day 7, and there were no significant differences between groups (*n* = 3, *P* > 0.05).

PEI is one of the most positively charged dense polymers, which have high transfection activity* in vitro* and moderate activity* in vivo*. One disadvantage of PEI is its nonbiodegradable nature and its toxicity* in vivo*, which limits its application in* in vivo* delivery [32–34]. There are conflicting associations between the gene delivery efficiency and PEI toxicity. The presence of pBMP2 triggered biochemical reactions which changed cell function and behavior and finally increased cell proliferation [35, 36]. MTT-test is a method of investigating the proliferation of cells. As showed in Figure 4, there was no difference for hPDLSCs proliferation among the core-shell scaffolds group, single axial scaffolds group, and control groups. We did not observe signs of PEI cytotoxicity during transfection; this could be due to the PEI amount used in our experiment, which was insufficient for an appreciable toxicity. The other possible reason is that the core-shell structure of the nanofibers limits direct contact with hPDLSCs.

**Figure 5:**
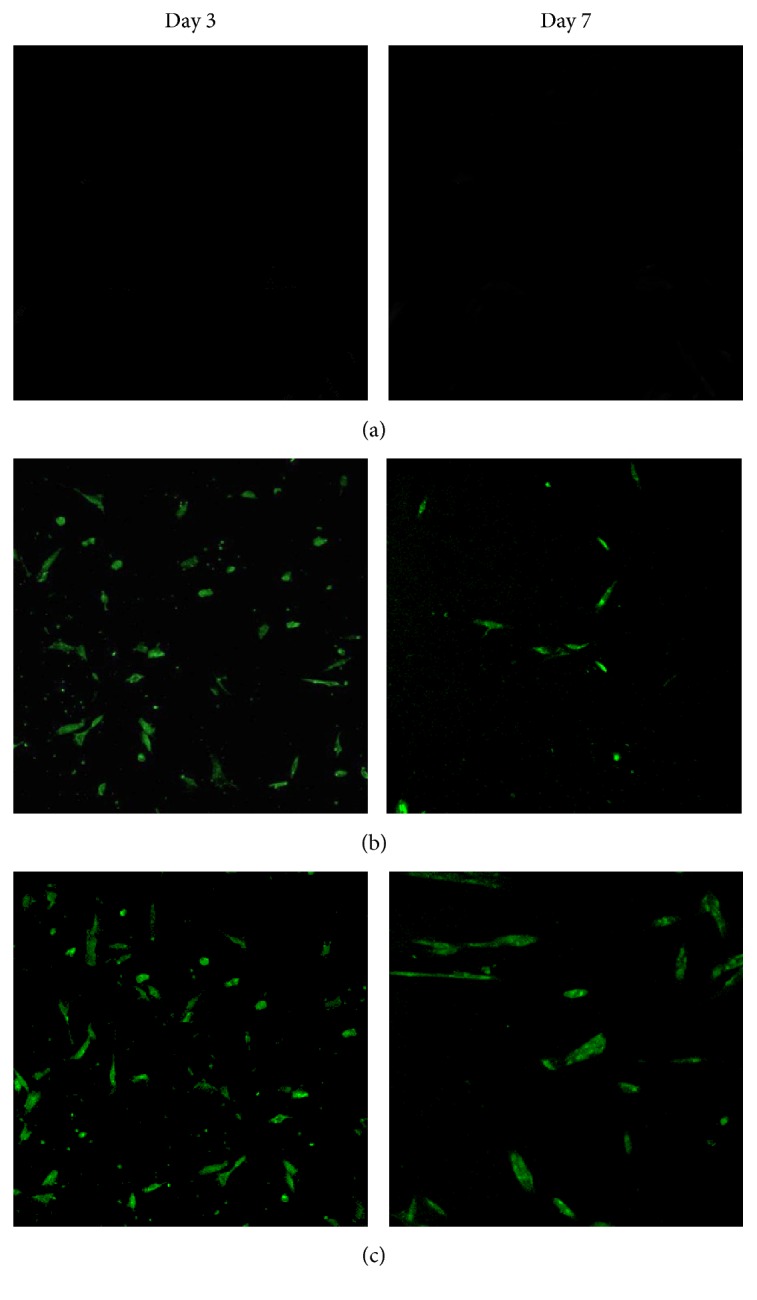
GFP expression in the three groups at days 3 and 7. (a) pBMP2 without PEI. (b) Single axial scaffolds. (c) Core-shell scaffolds.

**Figure 6:**
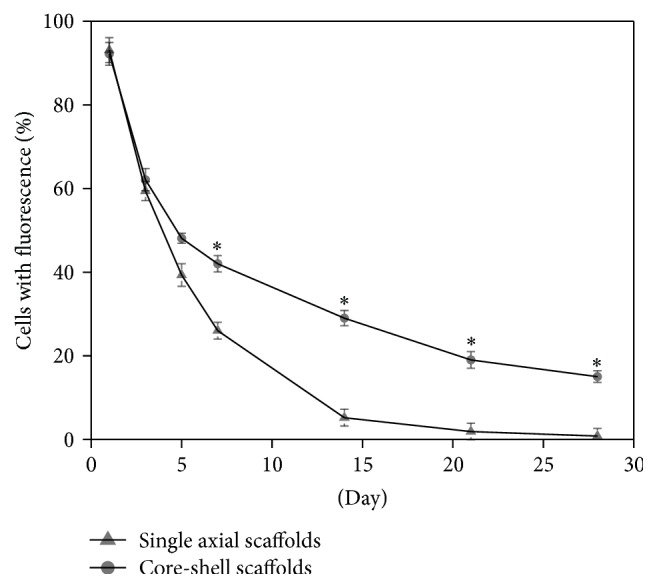
Cells fluorescence percentage after culture with core-shell scaffolds and single axial scaffolds at 1, 3, 5, 7, 14, 21, and 28 days (*n* = 3, ^*∗*^ *P* < 0.05).

**Figure 7:**
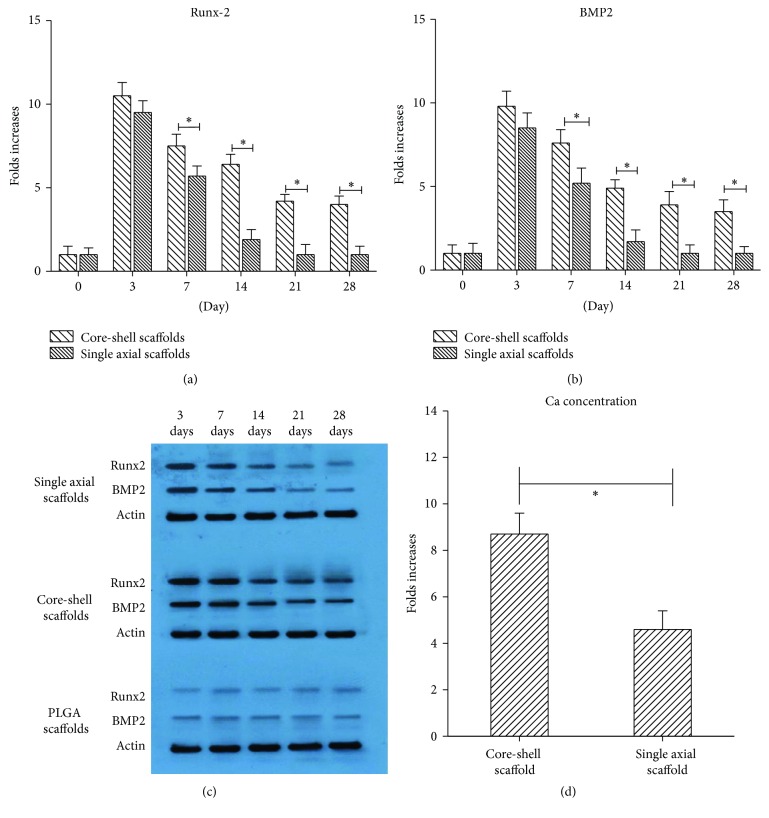
Runx2 (a) and BMP2 (b) mRNA expression was evaluated by qRT-PCR at several time points after transfection. (c) Runx2 and BMP2 protein expression evaluated by western blot. (d) Ca levels measured after 4-week culture (^*∗*^ *P* < 0.05, *n* = 3).

Similar to growth factor delivery, another important issue for gene delivery is to modulate transfection efficiency. To achieve gene transfection successfully, the effective concentration of target gene-vector complex should be released into the cell-surrounding microenvironment within an optimal time frame. Equivalent amounts of pBMP2 without PEI added directly to the cell medium revealed no green cells as showed in Figure 5, suggesting that there was no cellular transfection. The results were fairly accorded with experimental results reported in literature, in which the transfection efficiency of the PLGA scaffold with DNA without PEI is very low, less than 1% [22]. At an early time, the two scaffolds groups (the core-shell scaffolds and the single axial scaffolds) showed high transfection efficiency. Subsequently, the transfection efficiency of the single axial scaffold was reduced because of the pBMP2 concentration decrease in the nutrient due to its release. Compared to the single axial scaffolds, the core-shell scaffolds had significantly higher transfection efficiency for 7, 14, 21, and 28 days. The use of PEI/pBMP2 in combination with coaxial electrospinning enhanced the transfection efficiency.

The last important issue for gene delivery is the expression of the gene. The PEI/pBMP2 in hydrophilic core-layers followed by encapsulation with hydrophobic shell-layers in PLGA can prevent the direct contact of gene vectors with organic solvents. PEI/pBMP2 can be continuously released from the core-shell scaffolds, to avoid the PEI/pBMP2 complex burst release and then disassembled in the nutrient in a short time. The core-shell scaffolds could exhibit a long expression duration. Runx2/Cbfa1 is a global regulator of osteogenesis and is crucial for regulating the expression of bone-specific genes. Runx2 is a major target of the BMP pathway. BMP2 signaling stimulates Runx2 acetylation, increasing transactivation activity of Runx2 [37, 38]. Cbfa1/Runx2 and BMP2 mRNA expression was quantitatively evaluated by real-time PCR. Compared with the single axial scaffolds, Runx2 and BMP2 mRNA expression in the core-shell scaffolds increased significantly after 7 days (*P* < 0.05), although the pBMP quantity in the same area of the two scaffolds groups was not always the same. Assayed by western blotting, Runx2 and BMP2 protein expression also indicated that the hPDLSCs cultured with core-shell scaffolds clearly showed more protein expression and longer expression time. Calcification is another important indicator of osteoblast differentiation, which plays a major role in the formation of mineral deposits in the matrix during bone tissue engineering. As the cell messenger, its regularity of change can adjust a variety of physiological processes. Ca concentration was measured as a marker of degree of calcification and can indirectly reflect the degree of osteogenic differentiation, evaluating the osteogenic activity of cells [39, 40]. The present study can also show that the Ca concentration increase is more pronounced for the hPDLSCs cultured with the core-shell scaffolds compared with that cultured with the single axial scaffolds after 28 days (*P* < 0.05).

## 5. Conclusion

This study demonstrated that PEI/pBMP2 nanoparticles could be incorporated into a core-shell electrospun membrane, maintaining sustained release capability and biological activity. The prepared electrospun membrane showed satisfactory mechanical properties. Finally, the core-shell scaffold showed a good gene release behavior and prolonged expression time with high transfection efficiency. Therefore, our results could represent new insights to promote periodontal tissue regeneration.

## Figures and Tables

**Table 1:** Sense and antisense primers' sequences used to amplify gene transcripts by RT-PCR.

Gene	Sense sequence	Antisense sequence
Cbfa1/Runx2	ACA ACC ACA GAA CCA CAA G	TCT CGG TGG CTG GTA GTG A
BMP2	TAT GCT CGA CCT GTA CCG C	CAC TTC CAC CAC AAA CCC

**Table 2:** Mechanical properties of the three groups of electrospun scaffolds (mean ± standard deviation).

	Core-shell scaffold	Single axial scaffold	PLGA scaffolds
Ultimate strength (MPa)	3.51 ± 0.5	2.57 ± 0.31	4.60 ± 0.46
Elastic modulus (MPa)	11.34 ± 3.22	10.23 ± 4.08	15.57 ± 0.96
Ultimate strain (%)	29.87 ± 3.98	26.45 ± 2.53	35.46 ± 3.92
