# Corrigendum to “Fabrication of Core-Shell PEI/pBMP2-PLGA Electrospun Scaffold for Gene Delivery to Periodontal Ligament Stem Cells”

**DOI:** 10.1155/2017/7427606

**Published:** 2017-03-15

**Authors:** Qiao Xie, Lie-ni Jia, Hong-yu Xu, Xiang-gang Hu, Wei Wang, Jun Jia

**Affiliations:** ^1^State Key Laboratory of Military Stomatology and National Clinical Research Center for Oral Diseases and Shaanxi Key Laboratory of Oral Diseases, Department of Prosthodontics, School of Stomatology, The Fourth Military Medical University, Xi'an, Shaanxi 710032, China; ^2^No. 422 Hospital of PLA, Zhanjiang, Guangdong 524005, China; ^3^Hospital of PLA No. 93523 Unit, Yongji, Shanxi 044500, China

In the article titled “Fabrication of Core-Shell PEI/pBMP2-PLGA Electrospun Scaffold for Gene Delivery to Periodontal Ligament Stem Cells” [1], there was an error in the Acknowledgments section, which should be corrected as follows:

The authors would like to thank the financial support received from the National Science Foundation of China (no. 81271136). This investigation was supported by the School of Stomatology, Department of Military Toxicology, the Fourth Military Medical University.
